# Effects and Mechanisms of Polyunsaturated Fatty Acids on Age-Related Musculoskeletal Diseases: Sarcopenia, Osteoporosis, and Osteoarthritis—A Narrative Review

**DOI:** 10.3390/nu16183130

**Published:** 2024-09-16

**Authors:** Haoqi Chen, Ruogu Xiong, Jin Cheng, Jialu Ye, Yingzhen Qiu, Siyu Huang, Mengchu Li, Zhaoyan Liu, Jinzhu Pang, Xuguang Zhang, Shanshan Guo, Huabin Li, Huilian Zhu

**Affiliations:** 1 Department of Nutrition, School of Public Health, Sun Yat-sen University, Guangzhou 510080, China; chenhq55@mail2.sysu.edu.cn (H.C.); xiongrg@mail2.sysu.edu.cn (R.X.); chengj225@mail2.sysu.edu.cn (J.C.); yejlu@mail2.sysu.edu.cn (J.Y.); qiuyzh8@mail2.sysu.edu.cn (Y.Q.); huangsy9@mail2.sysu.edu.cn (S.H.); limch55@mail2.sysu.edu.cn (M.L.); liuzhy235@mail.sysu.edu.cn (Z.L.); lihuabin@mail.sysu.edu.cn (H.L.); 2 Mengniu Institute of Nutrition Science, Global R&D Innovation Center, Inner Mongolia Mengniu Dairy (Group) Co., Ltd., Hohhot 011050, China; pangjinzhu@mengniu.cn (J.P.); fxgzhang@mengniu.cn (X.Z.); guoshanshan@mengniu.cn (S.G.); 3 Sun Yat-sen University-Mengniu Joint Research Center of Nutrition and Health for Middle-Aged and Elderly, School of Public Health, Sun Yat-sen University, Guangzhou 510080, China

**Keywords:** polyunsaturated fatty acids, PUFAs, musculoskeletal disease, sarcopenia, osteoporosis, osteoarthritis

## Abstract

**Background:** The process of the globally aging population has been accelerating, leading to an increasing social burden. As people age, the musculoskeletal system will gradually go through a series of degenerative and loss of function and eventually develop age-related musculoskeletal diseases, like sarcopenia, osteoporosis, and osteoarthritis. On the other hand, several studies have shown that polyunsaturated fatty acids (PUFAs) possess various important physiological functions on the health of muscles, bones, and joints. **Objective:** This narrative review paper provides a summary of the literature about the effects and mechanisms of PUFAs on age-related musculoskeletal diseases for the prevention and management of these diseases. **Methods:** Web of Science, PubMed, Science Direct, and Scopus databases have been searched to select the relevant literature on epidemiological, cellular, and animal experiments and clinical evidence in recent decades with keywords “polyunsaturated fatty acids”, “PUFAs”, “omega-3”, “omega-6”, “musculoskeletal diseases”, “sarcopenia”, “osteoporosis”, “osteoarthritis”, and so on. **Results:** PUFAs could prevent and treat age-related musculoskeletal diseases (sarcopenia, osteoporosis, and osteoarthritis) by reducing oxidative stress and inflammation and controlling the growth, differentiation, apoptosis, and autophagy of cells. This review paper provides comprehensive evidence of PUFAs on age-related musculoskeletal diseases, which will be helpful for exploitation into functional foods and drugs for their prevention and treatment. **Conclusions:** PUFAs could play an important role in the prevention and treatment of sarcopenia, osteoporosis, and osteoarthritis.

## 1. Introduction

In recent years, the global population’s aging process has been irreversible, and it shows an accelerating trend. The World Health Organization (WHO) reported that in 2020, the population worldwide of 60 or older was around 1 billion, which accounts for 13.5% of the whole world population of 7.8 billion. By 2050, that figure is expected to approach 2.1 billion [1]. In the progression of aging, organisms undergo a series of gradual degenerative changes that lead to the accumulation of inflammation, the intensification of oxidative stress, apoptosis, and, thus, damage to the structure and function of cells and organs [2,3]. With aging, the risk of various age-related diseases increases, for instance, cardiovascular diseases, metabolic diseases, cancers, and musculoskeletal diseases. Among these diseases, age-related musculoskeletal diseases are a kind of diseases that, due to the longtime of mechanical pressure and biological changes, bones, muscles, and joints progressively experience a process of degeneration and eventually develop into a range of age-related musculoskeletal disorders, such as sarcopenia, osteoporosis, and osteoarthritis [4,5]. Globally, age-related musculoskeletal disorders are among the main causes of morbidity and death among the elderly, which also cause a huge economic burden. A total of 23.1% of the total of the Global Burden of Disease (GBD) is attributable to diseases in people aged 60 and over, and musculoskeletal diseases account for 7.5% of the total burden [6]. Therefore, improving the musculoskeletal function of the elderly has a vital part in improving their quality of life as they age.

**Figure 1:**
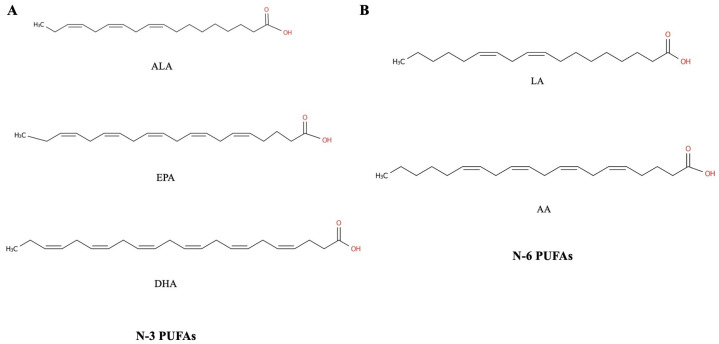
The chemical structures of several polyunsaturated fatty acids: (**A**) structures of *n*-3 PUFAs and (**B**) structures of *n*-6 PUFAs. AA—arachidonic acid; ALA—α-linolenic acid; DHA—docosahexaenoic acid; EPA—eicosapentaenoic acid; LA—linoleic acid; PUFA—polyunsaturated fatty acid.

Numerous studies have indicated that polyunsaturated fatty acids (PUFAs) offer certain benefits for cardiovascular diseases, diabetes, and other age-related diseases [7,8,9,10,11]. In particular, PUFAs are beneficial to the health of the musculoskeletal system and can improve age-related musculoskeletal diseases. According to the location of the first unsaturated bond, fatty acids with two or more unsaturated double bonds are referred to as PUFAs. These PUFAs can be further classified as omega-3, omega-6, omega-9, and so on. The *n*-3 family mainly consists of α-linolenic acid (ALA), eicosapentaenoic acid (EPA), and docosahexaenoic acid (DHA). The *n*-3 PUFAs take part in several biological processes, including oxidative energy supply and participation in biofilm construction; they have been proven to improve the body’s immune function, neuromuscular function, lipid distribution, and inflammation level [12,13,14]. The *n*-6 series mainly includes linoleic acid (LA), γ-linolenic acid, and arachidonic acid (AA). The *n*-6 PUFAs can regulate blood lipids, participate in phospholipid production, lower cholesterol, and promote growth and development [15,16] (Figure 1).

Moreover, LA and ALA are essential fatty acids (EFA), a class of fatty acids that are nutritionally essential but cannot be synthesized by mammals. The body can utilize EFA to synthesize longer-chain PUFAs. In most mammals, double bonds can be added at positions Δ4, Δ5, Δ6, and Δ9, but never beyond Δ9 [17,18]. On the other hand, some plants can synthesize EFAs by introducing double bonds at the Δ12 and Δ15 positions [19]. Therefore, the supplement of PUFAs is very necessary, and obtaining PUFAs directly from dietary food is the most efficient and important way.

**Figure 2:**
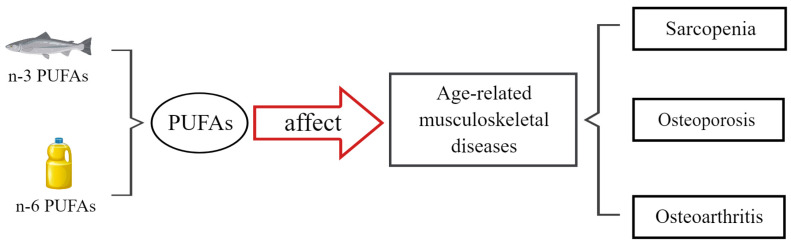
The effects of polyunsaturated fatty acids on age-related musculoskeletal diseases. PUFAs could protect against age-related musculoskeletal diseases, with a focus on sarcopenia, osteoporosis, and osteoarthritis. PUFA—polyunsaturated fatty acid.

Although the effects and mechanisms of PUFAs on sarcopenia, osteoporosis, and osteoarthritis have been widely studied, the relative information has not been comprehensively summarized and discussed. This paper reviews the studies in recent decades on the potential relationship between PUFAs and several age-related musculoskeletal diseases (sarcopenia, osteoporosis, and osteoarthritis). Then, we also discussed the underlying molecular mechanisms of the effect of PUFAs on these diseases (Figure 2). This review aims to provide a reference for managing and preventing these diseases.

## 2. Methods

We looked through the Web of Science, PubMed, Scopus, and Science Direct databases to select the relevant literature (including international articles written in English in recent decades, online reports, and textbooks). The keywords used in searching included “polyunsaturated fatty acids”, “PUFAs”, “omega-3”, “omega-6”, “musculoskeletal diseases”, “sarcopenia”, “osteoporosis”, “osteoarthritis”, and so on. The search step was completed in February 2024. Then, we reviewed the abstract of the articles to make sure they fit the topic of this review and removed all duplicates. Epidemiological, cellular, and animal experiments, as well as clinical evidence in recent decades on the effects and mechanisms of PUFAs on age-related musculoskeletal diseases, were summarized and discussed to integrate this narrative review. Because this is a narrative review paper, it does not need to record the literature search on any particular platform [20,21].

## 3. Sarcopenia

### 3.1. Prevalence of Sarcopenia

A syndrome known as sarcopenia is the age-related loss of strength and muscle mass, which is an important cause of the high incidence of fractures, some chronic diseases, and death [22,23,24]. Sarcopenia affects 10–23% of the elderly worldwide [25]. Oceania was observed with the highest prevalence (40%) applying the definitions established by the European Working Group on Sarcopenia in Older People (EWGSOP) [26], followed by South America (35%) utilizing muscle mass as standard, while Asia (15%) with the Asian Working Group for Sarcopenia (AWGS) [27], and Europe with EWGSOP2 [28] had the lowest prevalence (1%) [25]. In China, several community surveys in recent years have found an overall incidence of 11–19% [29,30,31]. Additionally, people with various illnesses have a higher prevalence of sarcopenia than the general population. For instance, the prevalence of individuals with diabetes was 18%, and the number of patients with unresectable esophageal cancer was up to 66% [32]. Therefore, many old people around the world are accompanied by muscle problems that seriously affect their quality of life.

### 3.2. Pathogenesis of Sarcopenia

Many factors can lead to sarcopenia in old people. Most current studies have shown that the pathogenesis of sarcopenia mainly includes muscle mitochondrial dysfunction, satellite cell loss and dysfunction, chronic inflammation, and related hormonal changes [33,34,35,36].

Mitochondrial dysfunction is a central mechanism of skeletal muscle senescence [33]. With bodies and cells aging, the oxidative phosphorylation capacity of skeletal muscle mitochondria is impaired, leading to the massive generation and accumulation of reactive oxygen species (ROS) in dysfunctional mitochondria [35]. However, skeletal muscle cells’ function mainly relies on oxidative metabolic pathways, which makes them very sensitive to ROS and very vulnerable to the deleterious effects of ROS production [37]. Ultimately, the abundant accumulation of mitochondrial ROS in skeletal muscle cells can accelerate telomere depletion as well as trigger cellular senescence, leading to sarcopenia [33]. 

One type of stem cell located in muscle tissue is called muscle satellite cell, and it has the ability to self-renew. Sarcopenia may be connected to the reduction in the number of satellite cells and the loss of the ability to regenerate, which are caused by aging [36,38]. In the aging satellite cells, the expression of several pathways, like Notch-p53 signaling axis and mammalian target of rapamycin complex 1 (mTORC1), which promote cell function and activity is decreased and dysregulated, while the associated inhibitory pathways are upregulated, such as Janus kinase-signal transducer and activator of transcription (JAK-STAT) and adenosine 5′-monophosphate (AMP)-activated protein kinase (AMPK)/p27 [39,40,41,42].

In the process of aging, the body presents a state of chronic inflammation. That is, various pro-inflammatory factors increase, and the levels of anti-inflammatory molecules decrease at the same time, which inhibits protein synthesis and promotes protein decomposition. This state of chronic inflammation causes anabolic imbalance in muscle tissue and involves multiple signaling pathways [43]. For example, an elevated level of interleukin-6 (IL-6) can cause myofibrillar protein loss and muscle atrophy. Tumor necrosis factor-α (TNF-α) can increase the decomposition of protein in skeletal muscle and reduce the production of muscle protein by transmitting ROS and activating the nuclear factor-κ-gene binding (NF-κB) signaling pathway [44,45]. 

### 3.3. Effects of PUFAs on Sarcopenia

In this section, the results from epidemiological studies are first summarized and discussed; then, the results of cellular and animal experiments are shown. Finally, the clinical evidence is provided.

Many studies have shown that PUFAs have been linked with better skeletal muscle growth and function, as well as a lower risk of sarcopenia. For instance, in some cross-sectional studies, omega-3 fatty acid consumption in older type 2 diabetic individuals was positively correlated with appendicular skeletal muscle mass index (ASMI) (odds ratio (*OR*) = 0.09; 95%confidence interval (*CI*) = 0.04, 0.06), step count (ρ = 0.524; *p* =  0.01), and grip strength (β = 0.757; *p* = 0.04) [46,47,48,49], and was negatively correlated with sarcopenia (*OR* = 0.29; 95% *CI* = 0.14, 0.60) [50]. In a case-control study, the EPA and DHA levels were lower in the sarcopenic patients than those in the control group (*p* = 0.003 and *p* = 0.014) [51]. 

**Figure 3:**
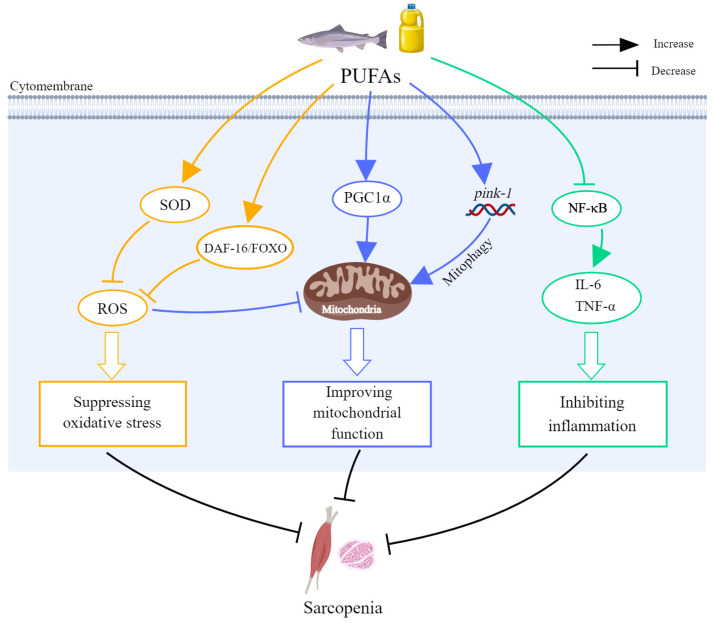
The effects and mechanisms of PUFAs against sarcopenia. PUFAs could increase the level of SOD and the expression of DAF-16/FOXO transcription factors in cells to inhibit the production of ROS, thus suppressing oxidative stress. PUFAs could also improve mitochondrial function by increasing the expression of the mitophagy gene *pink-1* and increasing PGC1α to promote mitophagy. PUFAs had the ability to suppress NF-κB activities, hence decreasing inflammatory markers like IL-6 and TNF-α. DAF-16—decay accelerating factor-16; FOXO—forkhead box O; IL-6—interleukin-6; NF-κB—nuclear factor-κ-gene binding; PGC1α—peroxisome proliferator-activated receptor γ coactivator 1α; PUFA—polyunsaturated fatty acid; ROS—reactive oxygen species; SOD—superoxide dismutase; TNF-α—tumor necrosis factor-α.

There are also some cell and animal experimental studies that showed PUFAs are beneficial to muscle health. PUFAs can ameliorate muscle damage caused by harmful factors. In an in vitro study, researchers used *Tunisian Pistacia lentiscus* L. seed oil (PLSO), rich in PUFAs and several nutrients with antioxidant properties, to counter the cytotoxic effects of 7β-hydroxycholesterol (7β-OHC) on mouse C2C12 myoblasts [52]. It was found that PLSO significantly attenuated the 7β-OHC-induced cytotoxicity, prevented organelle dysfunction, and lowered the oxidative stress level. The mechanism of this cytoprotective effect was mainly through lowering the level of ROS and increasing superoxide dismutase (SOD) and glutathione peroxidase (GPx) in cells, which restored mitochondrial function [52]. In another study, rat skeletal (L6) myotubes were cultured with saturated fatty acids (SFAs) either alone or together with a monounsaturated fatty acid (MUFA) or PUFA (linoleate, LO) [53]. The outcomes demonstrated that pro-inflammatory NF-κB, IL-6, and ROS levels rose, and peroxisome proliferator-activated receptor γ coactivator 1α (PGC1α) related to mitochondrial function decreased in SFA-induced L6 myotubes. LO can antagonize those changes, which means LO exhibits anti-inflammatory and antioxidant characteristics [53]. Moreover, a study used model animal *Caenorhabditis elegans* to explore the impact of LA on muscle. Their findings suggested that LA can repair mitochondrial function by weakening oxidative stress and promoting mitophagy, thereby improving skeletal muscle loss. The possible mechanism is increasing the expression of mitophagy genes *pink-1* and decay accelerating factor-16/forkhead box O (DAF-16/FOXO) transcription factors [54]. PUFAs also inhibit aging-related muscle loss and dysfunction. In a study, 75-week-old C57BL/6J mice received different diets (EPA-deprived or enriched diet) for 12 weeks to evaluate their efficacy in protecting against sarcopenia. The results indicated that the mice with an EPA-deprived diet showed lower grip strength, which can be improved by EPA supplementation [55]. This effect was probably caused by the transition of fiber type in skeletal muscle, which was a change in transcriptomic level [55]. In another study, 25-month-old Sprague Dawley (SD) rats were given gavage for 10 weeks of wheat oligopeptides and fish oil high in omega-3 PUFAs. According to the omics results, this study found that this combination dramatically improved muscle atrophy, oxidative stress, and inflammation levels in skeletal muscle and decreased aging-related muscle loss. The probable mechanism is that omega-3 PUFAs could promote protein synthesis and muscle regeneration [56]. The mechanisms of PUFAs in sarcopenia are shown in Figure 3 and Table 1.

Some randomized controlled trials (RCT) have tried to verify the improvement of PUFAs on skeletal muscle in aged people. For example, a study based on 200 older Chinese people showed that thigh circumference, total as well as appendicular skeletal muscle mass significantly increased (*p* < 0.001) after 6 months of fish oil capsules (1.34 g EPA + 1.07 g DHA/d) supplementation. Muscle strength and physical performance, including hand grip strength and timed up and go time (*p* < 0.001), were also improved [57]. In another study based on 94 healthy aged participants, the intervention with krill oil for 6 months promoted appendicular skeletal muscle mass, strength, and function, mainly including the vastus lateralis muscle thickness, grip strength, and knee extensor maximal torque (*p* < 0.05), which indicated that supplementation of krill oil could significantly increase muscle function and size [58]. Furthermore, a study found that 6 months of supplementing *n*-3 PUFA in healthy elderly not only exhibited a significant increase in muscle strength but also attenuated acute response to exercise without any effects on mitochondrial function [59]. Moreover, there was evidence that PUFAs, in combination with training, had a positive effect on sarcopenia. A RCT found that 24 weeks of *n*-3 PUFA-rich diet combined with resistance training stimulated the regional anti-inflammatory responses and growth responses in skeletal muscle by upregulating the expression of cell growth regulator (e.g., mTOR) and downregulating the expression of pro-inflammatory cytokine (e.g., IL-1β), which is advantageous to the skeletal muscle growth in active older women [60].

There are still some studies with inconsistent results. A study included 55 elderly patients with abdominal obesity with type 2 diabetes. The intervention group received 4 g/d of fish oil for 6 months, and the levels of serum EPA and DHA rose significantly, but there was no significant change in muscle mass [61]. The possible explanation for the different results was these subjects with type 2 diabetes had a long duration of insulin resistance and relatively older ages, so their resilience may be even worse, and their response to interventions may also be weaker. Another 3-year DO-HEALTH clinical trial showed that neither *n*-3 PUFAs supplemented alone nor combined with vitamin D or strength training improved the scores of short physical performance battery (SPPB) in a statistically significant way [62]. The reasons could be that 83% of participants in this study at baseline had been at moderate to high levels of physical activity. Therefore, the possibility of further benefit from extra exercise may be very slight. The details of clinical studies are shown in Table 2. 

## 4. Osteoporosis

### 4.1. Prevalence of Osteoporosis

Osteoporosis is a systemic bone disease, and its characteristics are reducing bone mineral density and impairing the microstructure of bone, which can lead to increased vulnerability and fracture susceptibility [71,72]. With the global elderly population expanding, the high-risk group of osteoporosis is also increasing, and fragile fractures have become a major obstacle to healthy aging.

The International Osteoporosis Foundation (IOF) estimated that 4 million men and 16 million women were affected by osteoporosis in six European countries (the UK, France, Germany, Italy, Spain and Sweden) [73]. In America, about 10 million Americans over 50 suffer from osteoporosis, and another 34 million are at risk [74]. In China, the overall prevalence of osteoporosis over 40 years old was 20.6% for women and 5.0% for men [75]. In addition, bone fraction is one of the most common complications of osteoporosis [76]. In 2010, about 21 million men and 137 million women aged 50 years and over worldwide reached the fracture threshold, and Asia had the top number of people over the threshold; the number is projected to double in the next 40 years [77]. As a result, osteoporosis and associated fractures have become a significant disease burden to society.

### 4.2. Pathogenesis of Osteoporosis

Osteoclasts are large, multinucleated cells attached to bone, and their main function is to release the enzymes associated with osteolysis for bone resorption [78,79]. Studies have shown that many factors, such as hormones, cytokines, inflammatory factors, and noncoding RNA, can act on the signaling pathways that promote the differentiation and maturation of osteoclasts, which result in enhanced bone resorption, bone loss, and osteoporosis [80]. The signaling pathways mainly includes IL-1/TNF-α, receptor activator of nuclear factor κB ligand/receptor activator of nuclear factor κB/osteoproteinogen (RANKL/RANK/OPG) and their downstream signaling pathways, mitogen-activated protein kinase (MAPK) cascade, NF-κB, protein kinase B (PKB), *c*-jun *N*-terminal kinase (JNK), and extracellular regulated protein kinases (ERK) [81,82,83,84].

Another key group of cells is osteoblasts, which contribute to forming bone and strengthening through the synthesis and secretion of collagen, as well as the formation of hydroxyapatite by mineralizing inorganic phosphorus and calcium ions [85]. The signaling molecules that play a crucial role in osteoblast turnover are Runx2, β-catenin, osterix, and their related signaling pathways, for instance, Wnt/β-catenin, Notch and bone morphogenetic protein (BMP)-Smad signaling pathways. Those molecules and signaling pathways regulate the growth, activation, and maturation of the osteoblasts, and they are essential for bone remodeling [86,87,88,89,90].

### 4.3. Effects of PUFAs on Osteoporosis

Accumulating studies have indicated that PUFAs could play an important role in maintaining bone health. *n*-3 PUFA intake was inversely associated with the risks of incident and recurrent fractures, and this benefit was more distinct in individuals with a higher genetic risk of fractures. In some cross-sectional studies, the BMD in the spine (β = 0.155; *p* = 0.009) and femur (β = 0.287; *p* = 0.043) showed a significant positive correlation with total plasma *n*-3 PUFA [91,92] and serum docosapentaenoic acid (DPA) had a positive correlation with BMD of the head (β = 0.002; *p* = 0.008) and lumbar spine (β = 0.001; *p* = 0.036) [93]. Logistic regression analysis also showed that higher levels of plasma *n*-3 PUFAs (*OR* = 0.751; *p* = 0.022) were protective factors for low bone mass [91]. Compared to patients with osteoarthritis, those with hip fractures had 26.2% lower plasma *n*-3 levels [94]. However, the nutritional pattern abundant in omega-6 PUFAs had a negative association with the BMD of the hip (*r* = −0.215, *p* < 0.05) [95]. In a Mendelian randomization study, omega-6 PUFAs were also inversely related to total body BMD (β = −0.052; *p* = 0.0106) [96]. In a cohort study, the risks of the total fractures (hazard ratio (*HR*) = 0.93; 95% *CI* = 0.89, 0.97), total recurrent fractures (*HR* = 0.88; 95% *CI* = 0.82, 0.96), vertebrae fractures (*HR* = 0.85; 95% *CI* = 0.72, 0.99), and hip fractures (*HR* = 0.83; 95% *CI* = 0.75, 0.92) were lower in those subjects who have the habit of using fish oil supplements [97]. Moreover, this negative association between fish oil supplementation and total fractures was stronger in individuals with higher fracture genetic risk scores [97]. 

Some studies showed that the level of PUFA metabolism in aging bone cells changes, and supplementation with PUFAs has many health benefits for bones. For example, in a study, researchers applied metabolomics to explore the change in the level of *n*-3 PUFA in aged osteoblasts and found that the amount of *n*-3 PUFA decreased in tandem with a reduction in the expression of genes associated with bone metabolism (e.g., RANKL/OPG, insulin-like growth factor-1, IGF-1), as well as increases in genes linked to aging and oxidative stress damage (e.g., MDA). Intake of *n*-3 PUFA improved aging-related osteoporosis by adjusting unsaturated fatty acid metabolism in senescent osteoblasts [98]. In another study, the non-polar lipid fraction of green shell mussel oil (GSM), which is high in long-chain omega-3 PUFA, was introduced into the culture of osteoclasts. The results showed that non-polar lipid from GSM oil reduced the tartrate-resistant acid phosphatase (TRAP) activity and the numbers of TRAP cell in a manner that is dependent on the dosage and the expression of some genes related to cell differentiation decreased, like nuclear factor of activated T-cells, cytoplasmic 1 (NFATc1), carbonic anhydrase II (CA II), cathepsin K (CTSK), and matrix metalloproteinase-9 (MMP-9). The non-polar lipid fraction of GSM oil had the effect of inhibiting osteoclastogenic activity [99]. 

**Figure 4:**
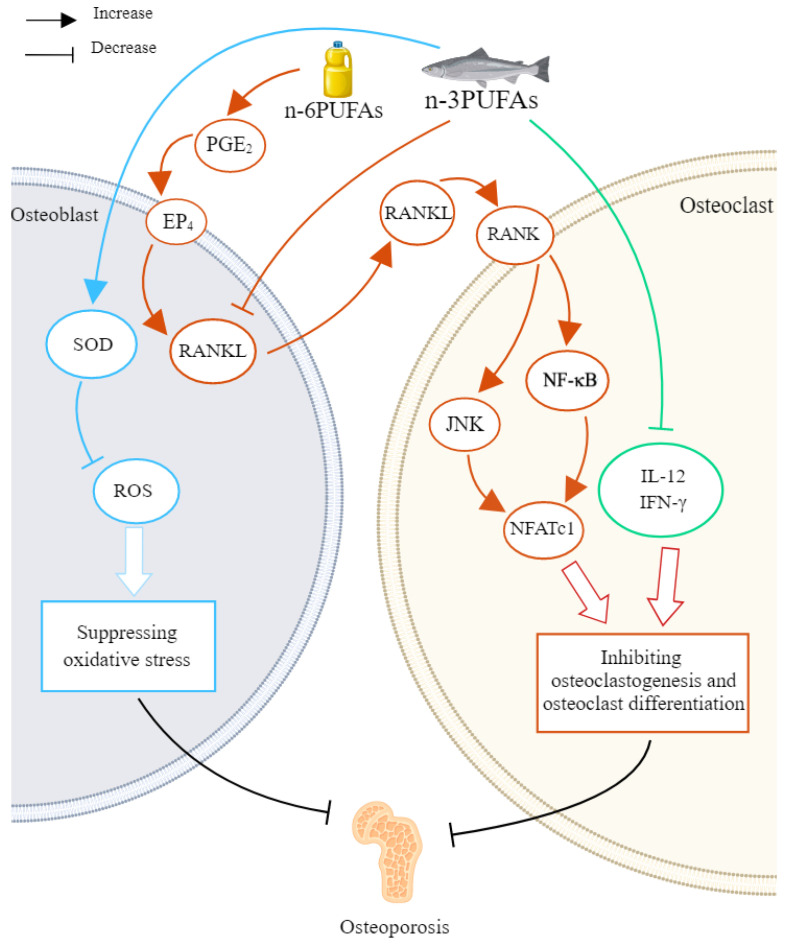
The effects and mechanisms of PUFAs against osteoporosis. *n*-3 PUFAs could raise the level of SOD to reduce ROS, thus suppressing oxidative stress in osteoblasts; *n*-3 PUFAs could reduce RANKL expression in osteoblasts, while *n*-6 PUFAs could raise the expression via increasing PGE_2_/EP_4_. RANKL could combine with RANK on osteoclast and then activate NF-κB and JNK pathways to ultimately stimulate NFATc1, the important transcription factor for the differentiation of osteoclast. *n*-3 PUFAs could increase the level of IFN-γ and IL-12, the strong suppressors of osteoclastogenesis. Osteoclastogenesis and osteoclast differentiation would improve bone resorption and bone loss, which led to osteoporosis. IFN-γ—interferon-γ; IL-12—interleukin-12; JNK—*c*-jun *N*-terminal kinase; NF-κB—nuclear factor-κ-gene binding; NFATc1—nuclear factor of activated T-cells cytoplasmic 1; PGE_2_—prostaglandin E_2_; PUFA—polyunsaturated fatty acid; RANK—receptor activator of nuclear factor κB; RANKL—receptor activator of nuclear factor κB ligand; ROS—reactive oxygen species; SOD—superoxide dismutase.

Several animal experiments showed that a diet high in *n*-3 PUFAs significantly improved bone accumulation and bone function. For example, in a study, 12-month-old mice were given 1% or 4% highly purified concentrated fish oil (CFO) diets for 12 months. The results showed that mice from the 4% CFO group maintained higher bone mineral density (BMD) in the process of aging [100]. The possible mechanisms of the protection included decreasing the levels of the bone resorption marker (e.g., TRAP and IL-6), reducing the stimulating factor RANKL without influencing its receptor OPG, increasing the suppressors of osteoclastogenesis (e.g., IL-12, interferon-γ, IFN-γ), and downregulating the inflammatory signaling pathways like NF-κB, JNK, and p38 MAPK [100]. In another study, supplementation of DHA in SD rats increased BMD and bone mineral content (BMC) of the whole body, lumbar spine, and long bone, and the bone cortical microstructure parameters and peak force of the lumbar spine were improved. Additionally, this study also found that during and after sexual maturation, intake of dietary DHA (0.1, 0.4, 0.8, and 1.2% *w*/*w*) would contribute to the peak bone mass, and higher doses of DHA had no further healthy benefit for bone [101]. Furthermore, adult Wistar rats in the group with diets that added flaxseed flour, which is rich in ALA, had greater total and spine BMD, total and spine BMC, and total bone area, as well as higher levels of osteocalcin. Flaxseed flour also improved the width of the diaphysis, BMD, maximum force, breaking strength, and stiffness of the femur [102]. However, *n*-6 PUFAs in the diet could be harmful to bone health. For instance, in a study, mice in the postmenopausal osteoporosis model were orally administered with krill oil or AA-rich oil diet. It found that AA diet inhibited BMD and the repair of trabecular microstructure. The underlying mechanism was that the AA diet upregulated the expression of RANKL mediated by prostaglandin E_2_/EP_4_ receptor, thereby enhancing the NF-κB pathway, which leads to bone resorption [103]. The mechanisms of PUFAs on osteoporosis are shown in Figure 4 and Table 3. It should be pointed out that the relationship between osteoporosis and *n*-3, *n*-6 PUFAs was inconsistent. That is, taking significant amounts of *n*-3 PUFAs in the diet was both healthy and safe, whereas too much *n*-6 PUFAs in the diet could be damaging to health.

Some clinical trial studies have also indicated that PUFAs, particularly *n*-3 PUFA, have positive effects on bone health. In a study, older postmenopausal women received 1.2 g/d of EPA/DHA for 6 months while the control group was provided olive oil. It was found that, in this short-term intervention, DHA levels in red blood cells (RBCs) increased, and osteocalcin and bone-specific alkaline phosphatase dropped in the *n*-3 PUFA group [63]. In an RCT, healthy Japanese adults received 7.0 mL/d of olive oil as a placebo or perilla (*Perilla frutescens*) seed oil (PO), which is rich in ALA. After 12 months of intervention, the mean of BMD increased, and the levels of serum TRACP5b decreased significantly in the PO group [64]. At the same time, compared to the placebo group, erythrocyte plasma membrane ALA levels and biological antioxidant potential/diacron reactive oxygen metabolites ratios significantly increased in the PO group. These findings suggested that 12 months of PO intake alleviated age-related BMD decline via increasing ALA levels and inhibiting bone resorption [64]. In another study, high-dose fish oil supplementation was given to postmenopausal breast cancer survivors for 3 months. The results indicated that the levels of total *n*-3 PUFAs, EPA, and DHA in serum increased, but the levels of *n*-6 PUFAs and *n*-6:*n*-3 PUFA ratio in serum decreased, and bone resorption was inhibited [65]. 

Several studies, especially long-term studies, showed no significant effect. For example, in a trial, 2.6 g/d marine *n*-3 PUFA supplement for 44 weeks was provided to adult kidney transplant recipients after the transplant, while olive oil was given to the control. It was found that, in the intention-to-treat analyses, there was no significant difference in Delta BMD of any skeletal site and trabecular bone score (TBS) between the two groups [66]. The probable reason for this inconsistent result could be that the study subjects were mainly Norwegian, famous for their high intake of fish, so more supplementation was unlikely to provide significant advantages because the number of participants had probably already surpassed the marine *n*-3 PUFA threshold that is ideal for bone. In another study, HIV-infected patients with hypertriglyceridemia were given 2 g/d of *n*-3 PUFA or fenofibrate for 24 months. The outcomes showed that, although the BMD in the femoral neck (FN) region decreased noticeably in both groups, there was no statistical difference in the change of BMD in the lumbar spine and FN between the two groups [67]. According to the study, an imbalance in the proportion of *n*-3 and *n*-6 PUFAs in patients‘ diets could have contributed to the different outcomes in the trial. At the same time, the sample size of this trial was small (30 subjects per arm), and many subjects were lost to follow-up. The details of clinical studies are shown in Table 2.

## 5. Osteoarthritis

### 5.1. Prevalence of Osteoarthritis

Osteoarthritis is a degenerative, non-inflammatory, and progressive active joint disease. The pathological degeneration of articular cartilage, as well as new bone formation in the joint margin and subchondral region, are the main characteristics [104]. As GBD reported, in 2017, the global age-standardized point prevalence of diagnosed hip and knee osteoarthritis was 3.75%, and the annual incidence was 0.18% [105,106]. The prevalence rate showed a 9.3% increase from 1990 to 2017 and a 13.25% increase from 1990 to 2019 [105,107]. The standardized incidence of overall osteoarthritis in 2017 was 6.8 per 1000 person-years, and the prevalence was 10.7% in the United Kingdom [108]. The prevalence of symptomatic knee osteoarthritis was 17% in the United States and 14.6% in China [109,110]. In lower middle- and low-income countries, including countries in East Asia, Pacific and South Asia, and Sub-Saharan Africa, the prevalence of osteoarthritis varied widely from 1.42% to 83.73% [111]. Moreover, the prevalence in the US, China, and India has greatly increased by 79.63%, 156.58%, and 165.75%, respectively, from 1990 to 2019 [107]. Thus, osteoarthritis is an increasingly common disease that affects many health outcomes worldwide.

### 5.2. Pathogenesis of Osteoarthritis

In the past few decades, osteoarthritis has been widely studied, but the complex pathological mechanism of osteoarthritis is still not very clear [112]. It once was thought that prolonged overload and biomechanical degradation of joints were the cause of the destruction of articular cartilage and consequent inflammation, which led to pains and stiffness of joints [113]. Nowadays, chondrocyte apoptosis, joint-related tissue lesions, inflammation, and metabolic factors are considered to be several factors that could cause osteoarthritis [112,114,115]. Apoptosis is a mode of highly regulated programmed cell death. In chondrocytes of osteoarthritis, apoptotic cells increased dramatically [116]. Several studies have indicated that the signaling pathways (e.g., the mitochondrial-mediated caspase-dependent pathways and the death receptor pathway) [112] and cytokines (e.g., TNF-α, IL-1β, and IL-6) [115,117,118] related to apoptosis were increased in chondrocytes of osteoarthritis.

Osteoarthritis is a joint disease involving the change of articular cartilage and subchondral bone, ligament, capsule, synovial joints, and muscles around the structure [119], especially the change of subchondral bone and synovium [112]. Subchondral bone sclerosis was probably one of the important causes of osteoarthritis. In both the early and late stages, a series of structural changes of subchondral bone were observed, including pore remodeling of subchondral bone, irregular bone mineralization in the matrix tissue, enhancement of subchondral bone density, and sclerosis [120,121,122]. Moreover, synovial hyperplasia, hypertrophy, fibrosis, and the level of synovial inflammation were related to the occurrence and development of osteoarthritis [123,124].

### 5.3. Effects of PUFAs on Osteoarthritis

Some epidemiologic studies showed that dietary intake of PUFAs had associations with disease progression in osteoarthritis. For example, in a study, inverse relations were found between total *n*-3 PUFAs and DHA in plasma with patellofemoral cartilage loss [125]. In another case-control study, 100 female participants with symptomatic primary knee osteoarthritis were matched with 100 apparently healthy women. The results found that PUFA intake had a negative correlation with the Western Ontario and McMaster Universities Osteoarthritis Index (WOMAC) (*r* = −0.163; *p* < 0.05) [126]. In a 48-month follow-up study of subjects with radiographic knee osteoarthritis, researchers observed that higher dietary intakes of PUFAs appeared to be associated with less radiographic progression (*HR* = 0.67; 95% *CI* = 0.51, 0.89) [127]. However, the result of a study showed that none of the serum EPA, other specific *n*-3 PUFAs, and *n*-6 PUFAs levels were associated with the risk of knee osteoarthritis or other osteoarthritis outcomes [128]. The possible reason was that the measured serum PUFA levels were temporary and might not be associated with longer-term results.

**Figure 5:**
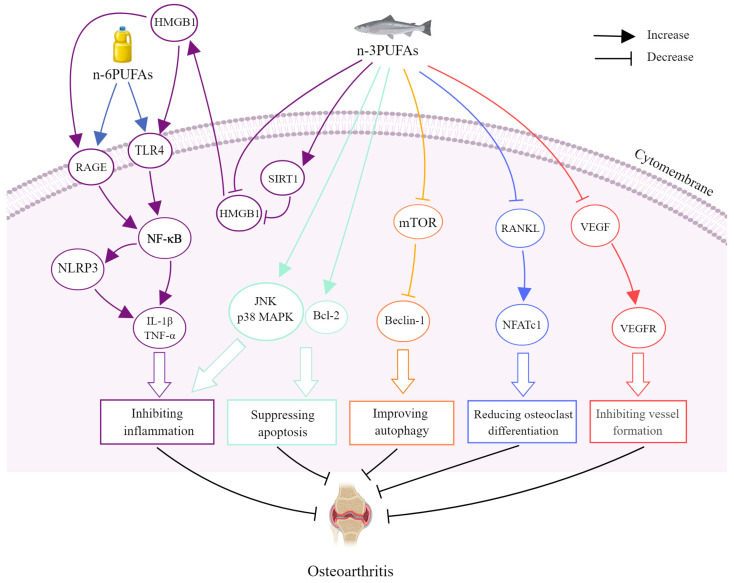
The effects and mechanisms of polyunsaturated fatty acids against osteoarthritis. *n*-3 PUFAs could increase SIRT1 and inhibit the HMGB1-RAGE/TLR4-NF-κB signaling pathway, while *n*-6 PUFAs could promote the pathway, which was associated with increasing the level of NLRP3 and inflammatory factors, like IL-1β and TNF-α. *n*-3 PUFAs could increasing JNK, p38 MAPK pathways expression and Bcl-2 to suppress apoptosis. *n*-3 PUFAs could raise the level of Beclin-1 by inhibiting the expression of the mTOR pathway to promote autophagy. *n*-3 PUFAs could lower the expression of RANKL and NFATc1 to suppress osteoclast differentiation and reduce VEGF and its receptor to inhibit vessel formation. Bcl-2—B-cell lymphoma-2; HMGB1—high-mobility group box 1; IL-1β—interleukin-1β; JNK—*c*-Jun *N*-terminal kinase; MAPK—mitogen-activated protein kinase; mTOR—mammalian target of rapamycin; NF-κB—nuclear factor-κ-gene binding; NFATc1—nuclear factor of activated T-cells cytoplasmic 1; NLRP3—NOD-like receptor protein 3; PUFA—polyunsaturated fatty acid; RAGE—receptor for advanced glycation end products; RANKL—receptor activator of nuclear factor κB ligand; SIRT1—sirtuin 1; TLR4—toll-like receptor 4; TNF-α—tumor necrosis factor-α; VEGF—vascular endothelial growth factor; VEGFR—vascular endothelial growth factor receptor.

Many experimental studies have investigated the beneficial effects of PUFAs on osteoarthritis and related mechanisms. For instance, in a study, human osteoarthritis chondrocytes and SD rats were both supplemented with DHA. It was discovered that rats with DHA had an increased collagen II-positive cell rate and thicker cartilage, while the rats also had a significantly lower Mankin score [129]. At the same time, in osteoarthritis chondrocytes, the expression of p-mTOR, p-JNK, p-p38, and the ratio of light chain 3-I/II (LC3-I/II) decreased, and the expression of Beclin-1 and B-cell lymphoma-2 (Bcl-2) increased. These outcomes indicated that DHA promoted proliferation, reduced apoptosis, and elevated autophagy of chondrocytes in osteoarthritis [129]. In another study, obesity-related post-traumatic osteoarthritis mice had 14 weeks of a diet that was abundant in *n*-3 PUFAs, and the diet alleviated the osteoarthritis-like lesions of articular cartilage and osteoarthritis progression, along with the reduction of proteins of high-mobility group box 1 (HMGB1), the receptor for advanced glycation end products (RAGE), and toll-like receptor 4 (TLR4) [130]. In SW1353 cells, DHA also significantly decreased HMGB1-RAGE/TLR4 signaling proteins, which were upregulated by IL-1β, while increasing the expression of sirtuin1. The overexpression of HMGB1 can reverse the inhibitory effect of DHA on the pathway [130]. In addition, *n*-6 PUFAs exacerbated obesity-related osteoarthritis, whereas *n*-3 PUFAs were protective against the disease by regulating the TLR4/NF-κB and NOD-like receptor protein 3 (NLRP3)/caspase-1/gasdermin D pathways [131]. Moreover, anterior cruciate ligament transection (ACLT)-induced rats were given DHA tail injection every other day. The results showed that the intervention group had less bone mass loss and angiogenesis, and in the osteochondral unit, the number of immunofluorescence-positive cells labeled with TRAP, RANKL, CD31, and endomucin decreased [132]. In the experiment of RAW264.7 cell, DHA inhibited TRAP-stained cells, area of bone resorption pits, and the mRNA expression of TRAP, CTSK, microphthalmia transcription factor (MITF), and NFATc1, while DHA also inhibited tube formation, proliferation, and migration, as well as vascular endothelial growth factor (VEGF)-C mRNA and vascular endothelial growth factor receptor2 (VEGFR2) protein expression in HUVECs [132]. The mechanisms of PUFAs on osteoarthritis are shown in Figure 5 and Table 4.

Clinical trials also have studied the health effects of PUFAs on joint pain and function. For example, in a trial, Japanese adults with mild knee pain as subjects were provided 2 g/d of krill oil for 30 days, and the symptoms of knee pain were assessed by the Japanese Knee Osteoarthritis Measure (JKOM) and Japanese Orthopedic Association score (JOA) [68]. After the intervention, in both the JKOM and JOA questionnaires, the results of the krill oil group showed a very big improvement, especially in the two questions of JKOM about knee pain and stiffness. Krill oil showed significant benefits on keen pain in sleeping and standing, as well as in the range of motion on both sides of the knees [68]. In another study, adults diagnosed with mild to moderate knee osteoarthritis or had regular knee pain were given 4 g/d of Krill oil as the intervention group for 6 months while the control group was given mixed vegetable oil. It was found that the krill oil group had greater improvements in knee stiffness, physical function, and knee pain scores [69]. 

Nevertheless, there is also a study that indicated that PUFAs did not improve joint diseases or function. In the Vitamin D and OmegA-3 Trial, participants of US older adults took marine omega-3 PUFAs supplementations (1 g/d Omacor^®^ + 840 mg/d EPA + DHA) for 3.8 to 6.1 years [70]. The results showed that during the follow-up period, at any point in time, the intervention group and placebo group between the WOMAC pain had no difference. Over time, marine omega-3 PUFA supplementations also did not significantly raise WOMAC function or stiffness scores, which indicated that a mean of 5.3 years of supplementing omega-3 PUFAs did not relieve the pain in the knee or improve function or stiffness in pain [70]. The reasons for this inconsistent result were that, firstly, the diagnosis of osteoarthritis was mainly based on self-report, which might be subject to bias; secondly, the dose of *n*-3 PUFAs was relatively lower [68,69]. The details of clinical studies are shown in Table 2.

## 6. Conclusions

Many studies showed that PUFAs could protect against age-related musculoskeletal diseases (sarcopenia, osteoporosis, and osteoarthritis), and the mechanisms were mainly reducing oxidative stress and inflammation and controlling the growth, differentiation, apoptosis, and autophagy of cells. However, some studies, especially several long-term studies, had inconsistent results. Therefore, in the future, more epidemiological studies with more reasonable research designs, larger sample sizes, and representative subjects should be conducted. In experimental studies, the underlying mechanisms of PUFAs on age-related musculoskeletal diseases should be explored further. Moreover, additional longer-term clinical trials ought to be carried out in order to validate the effects of PUFAs on age-related musculoskeletal diseases from the preclinical studies. Moreover, PUFAs could be exploited into functional foods and drugs for the prevention and treatment of age-related musculoskeletal diseases, sarcopenia, osteoporosis, and osteoarthritis. 

## Figures and Tables

**Table 1:** Effect of PUFAs on sarcopenia from cellular and animal experiments.

Study Type	PUFAs Type	Subject	Dose	Effects	Mechanisms	Ref.
In vitro	PLSO	7β-OHC-induced murine C2C12 myoblasts	100 μg/mL for 24 h	Prevented myoblast dysfunction and death Reduced oxidative stress	↑ SOD, GPx ↓ ROS, MDA, and ΔΨm	[52]
In vitro	LO	SFA-induced rat skeletal (L6) myotubes	100 mM for 16 h	Reduced inflammation and oxidation levels Improved mitochondrial function	↑ PGC1α ↓ ROS, IL-6, and NF-κB	[53]
In vivo	LA	*Caenorhabditis* *elegans*	50 μg/mL for 10 days	Improved skeletal muscle loss	↑ DAF-16/FOXO and *pink-1* ↓ ROS	[54]
In vivo	EPA	75-week-old C57BL/6J mice	1 wt% for 12 weeks, supplemented in diet	Suppressed aging-associated muscle dysfunction and muscle fiber type changes	Fast-to-slow fiber type transition; Muscle transcriptome alteration	[55]
In vivo	Fish oil	25-month-old SD rats	200, 400, 800 mg/kg for 10 weeks, oral gavage	Improved muscle atrophy, oxidative stress, and inflammatory levels and cell infiltration	Promoted protein synthesis and muscle regeneration	[56]

Abbreviations: 7β-OHC—7β-hydroxycholesterol; DAF-16—decay accelerating factor-16; EPA—eicosapentaenoic acids; FOXO—forkhead box O; GPx—glutathione peroxidase; IL-6—interleukin-6; LA—linoleic acid; LO—linoleate; MDA—malondialdehyde; NF-κB—nuclear factor-κ-gene binding; PGC1α—proliferator-activated receptor γ coactivator 1α; PLSO—Tunisian *Pistacia lentiscus* L. seed oil; ROS—reactive oxygen species; SD rat—Sprague Dawley rat; SFA—saturated fatty acid; SOD—superoxide dismutase; ↑—up regulation; ↓—down regulation.

**Table 2:** Clinical evidence of the effect of PUFAs on age-related musculoskeletal diseases.

Study Type	Intervention	Subject	Dose	Results	Ref.
Sarcopenia					
RCT	EPA + DHA	Older Chinese people	1.34 g EPA + 1.07 g DHA/d for 6 months	Increased mass, strength, and physical performance of muscle	[57]
RCT	Krill oil	Healthy elderly people	4 g/d for 6 months	Increased muscle thickness, grip strength, and knee extensor maximal torque	[58]
RCT	EPA + DHA	Healthy older adults	4 g/d for 6 months	Increased muscle strength Attenuated the acute response to exercise	[59]
RCT	*n*-3 PUFA-rich healthy diet + training	Older women	Fish and seafood intake ≥ 500 g/week for 24 weeks	Lowered the local level of inflammation Triggered growth responses in skeletal muscle	[60]
RCT	Fish oil	Type 2 diabetic patients with abdominal obesity	4 g/d for 6 months	Increased serum EPA and DHA levels but no significant change in muscle mass	[61]
RCT	Omega-3 PUFA	Adults aged 70 years or older	1 g/d for 3 years	Showed no significant increase in the scores of SPPB	[62]
Osteoporosis					
RCT	EPA/DHA	Older postmenopausal women	1.2 g/d for 6 months	Reduced bone turnover Improved RBC DHA levels in short-term supplementation	[63]
RCT	PO	Japanese adults	7.0 mL/d for 12 months	Had a positive effect on age-related BMD decline	[64]
RCT	Fish oil (EPA + DHA)	Postmenopausal breast cancer survivors	4 g/d for 3 months	Changed serum fatty acid levels Inhibited bone resorption	[65]
RCT	Marine *n*-3 PUFA	Adult kidney transplant recipients	2.6 g/d for 44 weeks	Showed no significant effect on promoting BMD	[66]
RCT	*n*-3 PUFA	HIV-infected patients	2 g/d for 24 months	Had no beneficial effect on BMD	[67]
Osteoarthritis					
RCT	Krill oil	Japanese adults	2 g/d for 30 days	Mitigated the pain and stiffness in knees	[68]
RCT	Krill oil	Adults with clinically diagnosed knee osteoarthritis or regular knee pain	4 g/d for 6 months	Improved keen pain, stiffness, and physical function	[69]
RCT	Marine omega-3 fatty acids	US older adults	1 g/d Omacor^®^ + 840 mg EPA + DHA for 3.8–6.1 years	Did not alleviate knee pain, stiffness, or enhance function	[70]

Abbreviations: BMD—bone mineral density; DHA—docosahexaenoic acid; EPA—eicosapentaenoic acid; PO—perilla seed oil; PUFA—polyunsaturated fatty acid; RBC—red blood cell; RCT—randomized controlled trial; SPPB—short physical performance battery; ↑—up regulation; ↓—down regulation.

**Table 3:** Effect of PUFAs on osteoporosis from cellular and animal experiments.

Study Type	PUFAs Type	Subject	Dose	Effects	Mechanisms	Ref.
In vitro	*n*-3 PUFAs	Osteoblasts	NA	Increased bone metabolism gene expression Decreased aging-related genes expression, oxidative stress damage	↑ RANKL/OPG, IGF-1 ↓ MDA, FOXO1	[98]
In vitro	GSM oil	RAW 264.7 osteoclasts	10–20 μg/mL for 48 h	Inhibited osteoclastogenic activity	↓ TRAP and NFATc1	[99]
In vivo	Fish oil	12-month-old C57BL/6 mice	1%, 4% for 12 months, supplemented in diet	Maintained higher BMD during aging	↑ BMD, IL-12, and IFN-γ ↓ TRAP5b, RANKL, and NF-κB	[100]
In vivo	DHA	SD rats	0.1, 0.4, 0.8, 1.2% *w*/*w* for 10 weeks, supplemented in diet	Increased bone mass, bone strength Improved trabecular microarchitecture	↑ BMC, BMD	[101]
In vivo	Flaxseed flour	Adult Wistar rats	25 g/100 g diet for 6 months	Produced greater BMD and femur resistance	↑ BMD, BMC, and osteocalcin	[102]
In vivo	AA	Ovariectomized mice	220 mg/kg for 3 months, oral administration	Impaired trabecular microstructure repair and BMD	↑ PGE_2_, RANKL, and NF-κB ↓ BMD	[103]

Abbreviations: AA—arachidonic acid; BMC—bone mineral content; BMD—bone mineral density; DHA—docosahexaenoic acid; FOXO—forkhead box O; GSM—green shell mussel oil; IFN-γ—interferon-γ; IGF-1—insulin-like growth factor-1; IL-12—interleukin-12; MDA—malondialdehyde; NA—not available; NF-κB—nuclear factor-κ-gene binding; NFATc1—nuclear factor of activated T-cells cytoplasmic 1; OPG—osteoproteinogen; PGE_2_—prostaglandin E_2_; PUFA—polyunsaturated fatty acid; RANKL—receptor activator of nuclear factor κB ligand; SD rat—Sprague Dawley rat; TRAP—tartrate-resistant acid phosphatase; ↑—up regulation; ↓—down regulation.

**Table 4:** Effect of PUFAs on osteoarthritis from cellular and animal experiments.

Study Type	PUFAs Type	Subject	Dose	Effects	Mechanisms	Ref.
In vitro	DHA	Human osteoarthritis chondrocyte	50 μg/mL for 1 h	Promoted chondrocyte proliferation Suppressed apoptosis and elevated autophagy	↑ Beclin-1 and Bcl-2 ↓ p-JNK, p-p38, p-mTOR, and LC3-I/II ratio	[129]
In vivo	DHA	SD rats	5 g/kg for 6 weeks, supplemented in diet		↑ Collagen II–positive cell rate ↓ Mankin score	[129]
In vitro	DHA	SW1353 cells	10 μM for 24 h	Alleviated osteoarthritis progression	↑ SIRT1 ↓ HMGB1, RAGE, TLR4, and Caspase-8	[130]
In vivo	Fish oil	Obesity-related post-traumatic osteoarthritis mice	8.4% *w*/*w* for 14 weeks, supplemented in diet			[130]
In vitro	*n*-3/*n*-6 PUFAs	SW1353 cells	NA	*n*-6 PUFAs exacerbated obesity-related osteoarthritis *n*-3 PUFAs were protective	*n*-6: ↑ TLR4, NF-κB, and NLRP3 *n*-3: ↓ TLR4, NF-κB, and NLRP3	[131]
In vivo	*n*-3/*n*-6 PUFAs	Obesity-related post-traumatic osteoarthritis mice				[131]
In vitro	DHA	RAW264.7 cells	NA	Protected cartilage by inhibiting the ability of bone remodeling and angiogenesis	↓ CTSK, TRAP, NFATc1, MITF, VEGF-C, VEGF-A, and VEGFR2	[132]
In vivo	DHA	ACLT-induced rats	1 mg/kg every other day for 2 months, injected in tail		↓ RANKL, CD31, and endomucin	[132]

Abbreviations: ACLT—anterior cruciate ligament transection; Bcl-2—B-cell lymphoma-2; CTSK—cathepsin K; DHA—docosahexaenoic acid; HMGB1—high-mobility group box 1; JNK—*c*-Jun *N*-terminal kinase; LC3—light chain 3; MITF—microphthalmia transcription factor; mTOR—mammalian target of rapamycin; NA—not available; NF-κB—nuclear factor-κ-gene binding; NFATc1—nuclear factor of activated T-cells cytoplasmic 1; NLRP3—NOD-like receptor protein 3; PUFA—polyunsaturated fatty acid; RAGE—receptor for advanced glycation end products; RANKL—receptor activator of nuclear factor κB ligand; SD rat—Sprague Dawley rat; SIRT1—sirtuin 1; TLR4—toll-like receptor 4; TRAP—tartrate-resistant acid phosphatase; VEGF—vascular endothelial growth factor; VEGFR—vascular endothelial growth factor receptor; ↑—up regulation; ↓—down regulation.
